# A Narrative Review on Fanconi Anemia: Genetic and Diagnostic Considerations

**DOI:** 10.1055/s-0042-1751303

**Published:** 2022-09-05

**Authors:** Preksha Sharma, Neha Sharma, Dhruva Sharma

**Affiliations:** 1 Department of Anatomy, SMS Medical College and Attached Hospitals, Jaipur, Rajasthan, India; 2 Department of Pharmacology, SMS Medical College and Attached Hospitals, Jaipur, Rajasthan, India; 3 Department of Cardiothoracic and Vascular Surgery, SMS Medical College and Attached Hospitals, Jaipur, Rajasthan, India

**Keywords:** Fanconi anemia, pancytopenia, acute myeloid leukemia, stress cytogenetics

## Abstract

Fanconi anemia (FA) is an autosomal recessive disorder, both genetically and phenotypically. It is characterized by chromosomal instability, progressive bone marrow failure, susceptibility to cancer, and various other congenital abnormalities. It involves all the three cell lines of blood. So far, biallelic mutations in 21 genes and one x-linked gene have been detected and found to be associated with FA phenotype. Signs and symptoms start setting in by the age of 4 to 7 years, mainly hematological symptoms. This includes pancytopenia, that is, a reduction in the number of white blood cells (WBCs), red blood cells (RBCs), and platelets. Therefore, the main criteria for diagnosis of FA include skeletal malformations, pancytopenia, hyperpigmentation, short stature, urogenital abnormalities, central nervous system, auditory, renal, ocular, and familial occurrence. Patients showing signs and symptoms of FA should be thoroughly evaluated. A complete blood count will reveal a reduced number of RBC, WBC, and platelets, that is, pancytopenia. Chromosomal breakage study/stress cytogenetics should be done in patients with severe pancytopenia. Momentousness timely diagnosis of current disease, prenatal diagnosis, and genetic counseling should be emphasized.

## Introduction

Fanconi anemia (FA) is an autosomal recessive disorder, both genetically and phenotypically. 1 It comes under the category of rare disease with a prevalence of 1 in 160,000 ( www.orpha.net ), but this is the most commonly inherited bone marrow failure syndrome. 2 Ashkenazi Jews, Afrikaners, and Spanish Gypsies have reported a higher incidence of FA. 3 It is characterized by chromosomal instability, progressive bone marrow failure, susceptibility to cancer, and various other congenital abnormalities. 4 It involves all the three cell lines of blood. In around 2% of the cases, it is inherited in an X-linked recessive manner. The mutations can be homozygous as well as heterozygous. 4 5 Out of all the phenotypes that have been reported to be associated with FA, hypersensitivity to DNA crosslinking agents like mitomycin C (MMC) and diepoxybutane (DEB) remains the most persistent one. 6

## Genetics

It is an autosomal recessive disorder except when there is the mutation of FANCB, which is located on the X chromosome, and inherited in an autosomal dominant manner when there is the mutation of FANCR. 7 8 A DNA sequence can be reckoned as an FA gene when an inactivating mutation is associated with chromosomal breakage when subjected to DNA cross-linking agents (such as MMC and DEB) in minimum one patient, and the breakage gets corrected by complementation with the wild-type version of the mutant alleles. 9 So far biallelic mutations in 21 genes and one x-linked gene have been detected and found to be associated with FA phenotype. 9 10 The genes that commensurate with different complementation groups in FA are involved in the FA/BRCA DNA damage repair pathway and play an important role in generating a response to DNA alkylating agents. 11 FANCA, FANCG, and FANCC have been found to be common FA complementation groups. 12 In around 60 to 70% patients diagnosed with FA, FANCA gene mutation predominates. These mutations may be insertion or deletion which may lead to premature termination or large deletions and null mutations. 13

### Multimer Complex by Fanconi Anemia Proteins

It is suggested that FA genes interact by the way of a biochemical pathway or multimer complex. Based on various findings which propound that FANCA and FANCC together form a complex and migrate to the nucleus, some investigators suggest that FANCC and FANCA do not interact with each other as they are in different compartments in the cell. Nuclear assembly of the protein complex, as well as binding, gets disrupted in FA-A, FA-C, FA-B, -E, -F, and -G cells. On the contrary, these remain intact in FA-D cells. 13 14 15 16 17 18 FANCA and FANCC form a complex and translocate to the nuclei, where they are involved in nuclear processes, such as DNA replication, DNA repair, and transcription. However, FANCD is a downstream gene and functions independently. Many studies concluded that FANCA, FANCC, and FANCG protein complexes are present in both nuclei and cytoplasm.

## Spindle Assembly Checkpoints

Spindle-assembly checkpoint was first discovered in yeast *Saccharomyces cerevisiae* . It is a signaling network which promotes tumor suppression by segregating high fidelity chromosomes during mitosis. This further helps in preventing aneuploidy. 19 20 21 22 The genes which have been identified are MAD (mitotic-arrest deficient) genes MAD1, MAD2, and MAD3 (BUBR1 in humans) and the BUB (budding uninhibited by benzimidazole) gene BUB1. 23 24 All these genes are found to be actively involved in a pathway which impedes the premature separation of sister chromatid, and this pathway is active in prometaphase. 23

## Signs and Symptoms Associated with Fanconi Anemia

Signs and symptoms start setting in by the age of 4 to 7 years, mainly hematological symptoms. This includes pancytopenia, that is, a reduction in the number of white blood cells (WBCs), red blood cells (RBCs), and platelets. Therefore, the main criteria for diagnosis of FA include skeletal malformations, pancytopenia, hyperpigmentation, short stature, urogenital abnormalities, central nervous system, auditory, renal, ocular, and familial occurrence. It has been observed that the incidence of congenital abnormalities in probands is more than in their affected sibling. Proband is a person who is first affected in the family and serves as the starting point for the genetic study in that family. 24 Chest pain, shortness of breath, dizziness, and fatigability are commonly present in FA. Patients give the history of petechiae, epistaxis, and unstoppable bleeding from a wound site that is due to thrombocytopenia. Flu-like illness and fever are frequently encountered and there is an increased risk of infection due to leukopenia.

Around 75% of FA patients present with birth defects. In approximately 50% of the cases, the child may present with short stature and café-au-lait spots (area of hyperpigmentation found on skin). 25 Other abnormalities of the extremity associated with FA are hypoplastic or absent radii, hypoplastic thumb (radial ray defect), and dysplastic ulna in the upper extremity, whereas hip dislocation, short toes, club foot, polydactyly, and thigh osteoma are seen in the lower extremity. Other skeletal abnormalities include frontal bossing, microcephaly and hydrocephaly, spina bifida, webbed and short neck, micrognathia, scoliosis, extra vertebrae, and abnormal ribs. Tracheoesophageal fistula, imperforate anus, umbilical hernia, Meckel's diverticulum are some of the gastrointestinal abnormalities encountered in FA. Gastrointestinal abnormalities are less common.

Physical manifestations of pancytopenia are bruising, petechiae, pallor, and coldness of hands and feet. FA patients develop bone marrow aplasia and pancytopenia between 2and 13 years of age. Some patients may present with these manifestations later in life, that is, during adolescence or even after that. Most of the FA patients eventually develop acute myeloid leukemia (AML) and myelodysplastic syndrome (MDS). 26 27 28 Therefore, regular follow-up of these patients is required. These patients are at a very high risk of developing hepatocellular carcinoma as well as hepatic adenoma. 29 30 In an analysis conducted by the European Fanconi Anemia Research Group, it was found that 8 and 9% of the patients diagnosed with FA developed MDS and AML, respectively. In some of the patients, the first hematological abnormality to be detected was AML/MDS and few patients presented with AML/MDS post bone marrow aplasia. Significantly younger patients presented in the former group as compared with the latter. Deletions of 5q, 7q, 20q, trisomy 8, monosomy 7, and chromosome1 abnormalities are frequently occurring cytogenetic abnormalities in FA patients with AML/MDS. 28

Growth abnormality which includes short stature is often associated with hormone deficiencies such as insulin resistance or deficiency, growth hormone deficiency, and pituitary hypofunction and hypogonadism. Hypogonadism is encountered in both sexes. There may be an underlying hypothalamic–pituitary dysfunction which leads to abnormal growth hormone secretion. Hence, endocrine evaluation for all children should be advised at an early age, in an order to improve quality of life and final height, by correcting growth and thyroid hormone deficiency. 1

## Differential Diagnosis

A large number of diseases resemble FA. All other hematological disorders which manifest signs and symptoms of FA and all the congenital structural disorders which are associated with FA should be ruled out.

**Paroxysmal nocturnal hemoglobinuria (PNH):** patients present with anemia, hemoglobin in urine, jaundice, and increased risk of thrombosis. PNH occurs due to the mutation of a gene ( *PIGA* ) which encodes for the protein glycosylphosphatidylinositol (GPI), in hematopoietic progenitor cells. This leads to the activation of complement system which further causes nocturnal hemolysis. 5

**Acquired aplastic anemia:** numerous toxicogenic agents lead to acquired hematopoietic stem cell destruction in the bone marrow. Hypocellularity of bone marrow without chromosome breakage, when chromosomes are subjected to stress cytogenetic test, is seen in aplastic anemia. However, pancytopenia along with chromosome fragility is the principal feature of FA. 5 30 31

**Diamond Blackfan anemia:** it is a pure red cell aplasia which typically presents as a defect in erythropoiesis. Patients present with macrocytic-normochromic anemia, normal platelets, normal WBCs, and low reticulocytes. 32

**Shwachman-Diamond syndrome (SDS):** impaired hematopoiesis, pancreatic insufficiency, and predisposition to leukemia are cardinal features of SDS. It is primarily bone marrow aplasia in which neutropenia predominates with the neutrophil count less than 1,500 × 10 ^9^ /L. 33

**Bloom syndrome:** patients present with photosensitivity, short stature, learning difficulties, telangiectatic erythema, malignancy, immunodeficiency, type 2 diabetes mellitus, and lupus-like skin lesions on the face. There is severe pre- and postnatal growth retardation. 34 35

**Congenital amegakaryocytic thrombocytopenia:** it predominantly affects platelets and leads to the absence of megakaryocytes. The patient presents with bleeding from day 1 of life or in the first month. There is severe thrombocytopenia which can later progress into leukemia or aplastic anemia. 36

**Table:** 

Differential diagnosis	Salient features
PNH	Patients present with anemia, hemoglobin in urine, jaundice, and an increased risk of thrombosis. PNH occurs due to the mutation of a gene ( *PIGA* ) which encodes for the protein GPI, in hematopoietic progenitor cells. This leads to the activation of complement system which further causes nocturnal haemolysis. 5
Acquired aplastic anemia	Numerous toxicogenic agents lead to acquired hematopoietic stem cell destruction in the bone marrow. Hypocellularity of bone marrow without chromosome breakage, when chromosomes are subjected to stress cytogenetic test is seen in aplastic anemia. However, pancytopenia along with chromosome fragility is the principal feature of FA. 5 30 31
Diamond Blackfan anemia	It is a pure red cell aplasia which typically presents as a defect in erythropoiesis. Patients present with macrocytic-normochromic anemia, normal platelets, normal WBCs, and low reticulocytes. 32
SDS	Impaired hematopoiesis, pancreatic insufficiency, and predisposition to leukemia are cardinal features of SDS. It is primarily bone marrow aplasia in which neutropenia predominates with the neutrophil count less than 1,500 × 10 ^9^ /L. 33
Bloom syndrome	Patients present with photosensitivity, short stature, learning difficulties, telangiectatic erythema, malignancy, immunodeficiency, type 2 diabetes mellitus, and lupus-like skin lesions on the face. There is severe pre- and postnatal growth retardation. 34 35
Congenital amegakaryocytic thrombocytopenia	It predominantly affects platelets and leads to the absence of megakaryocytes. Presents with bleeding from day 1 of life or in the first month. There is severe thrombocytopenia which can later progress into leukemia or aplastic anemia. 36

## Evaluation of Fanconi Anemia Patients

It was first propounded by Schroeder et al that chromosomal breakage study should be used as a cellular marker for FA patients. But later, many studies revealed that the hypersensitivity of FA cells to clastogens proves to be an authentic marker for the diagnosis of the disease. It is concluded from different studies that the average age of diagnosis of FA is 7 years. It is delayed till the time bone marrow failure sets in. However, prenatal diagnosis and increased awareness have led to an early diagnosis of the disease which further prevent severe complications. 5 37

Patients showing signs and symptoms of FA should be thoroughly evaluated. A complete blood count which reveals a reduced number of RBC, WBC, platelet is pancytopenia. There could be an increased fetal hemoglobin level due to high stress. Macrocytosis is depicted by increased mean corpuscular volume. Bone marrow biopsy and investigation show hypocellularity, absence of erythroid, myeloid, megakaryocytic cell lines, and bone marrow aplasia along with fatty marrow.

Chromosomal breakage study/stress cytogenetics should be done in patients with severe pancytopenia. Severe pancytopenia is when hemoglobin is less than 10 g/dL, reticulocyte count is less than 40,000/m, absolute neutrophil count is less than 100/mL, platelet count is less than 50,000/mL, and bone marrow cellularity is less than 25%. This should be done with DNA cross-linking agents (clastogens) such as MMC and DEB. These agents will increase the chromatid's gap, breaks, rearrangement, or reduplication. For those patients who have had hematopoietic stem cell transplants and patients who test negative for chromosomal breakage test with blood, cultured fibroblasts should be used. 1 5 Prenatal diagnosis of FA has a vital role to play. It should be known to the clinicians that amniocentesis, chorionic villous sampling, and umbilical cord blood sampling can be used for chromosomal breakage study. 1 38 39 40 Prenatal diagnosis of FA has played an important role in developing a model for umbilical cord blood transplant which can serve as a substitute to bone marrow transplant in the treatment of various hematological disorders. 1 41

Cell cycle analysis by flowcytometry can be used as an alternative diagnostic tool in differentiating between FA and non-FA I individuals. It is evident that the duration of G2M phase is prolonged in FA cells as compared with non-FA cells. Gene sequencing is recommended in those patients who test positive for chromosomal breakage tests. 42

## Conclusion

Well-timed diagnosis is of utmost importance in any genetic disease. Genetic counseling plays an important role in explaining the patients' relatives the course of the disease and also to prevent the occurrence of the same disease in the next child. The clinician must be well acquainted with the fact that timely diagnosis and genetic counseling are essential to start the therapy for the patient as early as possible. Parents of the child should be counseled regarding the availability of prenatal diagnosis and preimplantation genetic diagnosis. This will further help in reducing the burden of genetic diseases in society.
